# Partial Regulatory T Cell Depletion Prior to Schistosomiasis Vaccination Does Not Enhance the Protection

**DOI:** 10.1371/journal.pone.0040359

**Published:** 2012-07-03

**Authors:** Xuefeng Wang, Fan Liu, Sha Zhou, Zhipeng Xu, Jason Hoellwarth, Xiaojun Chen, Lei He, Rongbo Zhang, Feng Liu, Jun Wang, Chuan Su

**Affiliations:** 1 Department of Pathogen Biology and Immunology, School of Medicine, Anhui University of Science and Technology, Huainan, Anhui, China; 2 Department of Basic Medical Sciences, Medical College, Xiamen University, Xiamen, Fujian, China; 3 Department of Pathogen Biology and Immunology, Jiangsu Key Laboratory of Pathogen Biology, Nanjing Medical University, Nanjing, Jiangsu, China; 4 Department of Educational Affairs, Keck School of Medicine, University of Southern California, Los Angeles, California, United States of America; University of Southern California, United States of America

## Abstract

CD4^+^CD25^+^ regulatory T cells (Tregs) do not only influence self-antigen specific immune responses, but also dampen the protective effect induced by a number of vaccines. The impact of CD4^+^CD25^+^ Tregs on vaccines against schistosomiasis, a neglected tropical disease that is a major public health concern, however, has not been examined. In this study, a DNA vaccine encoding a 26 kDa glutathione S-transferase of *Schistosoma japonicum* (pVAX1-Sj26GST) was constructed and its potential effects were evaluated by depleting CD25^+^ cells prior to pVAX1-Sj26GST immunization. This work shows that removal of CD25^+^ cells prior to immunization with the pVAX1-Sj26GST schistosomiasis DNA vaccine significantly increases the proliferation of splenocytes and IgG levels. However, CD25^+^ cell-depleted mice immunized with pVAX1-Sj26GST show no improved protection against *S. japonicum*. Furthermore, depletion of CD25^+^ cells causes an increase in both pro-inflammatory cytokines (e.g. IFN-γ, GM-CSF and IL-4) and an anti-inflammatory cytokine (e.g. IL-10), with CD4^+^CD25^-^ T cells being one of the major sources of both IFN-γ and IL-10. These findings indicate that partial CD25^+^ cell depletion fails to enhance the effectiveness of the schistosome vaccine, possibly due to IL-10 production by CD4^+^CD25^-^ T cells, or other cell types, after CD25^+^ cell depletion during vaccination.

## Introduction

Schistosomiasis is one of the most important neglected tropical diseases (NTDs) and remains a major public health problem in endemic countries [1], [2]. Although schistosomiasis can be treated with praziquantel [3], the high re-infection rate limits the overall success of drug therapies [4], [5]. Therefore, the development of a safe, effective vaccine would significantly improve the long-term management of schistosomiasis and improve the efficacy of chemotherapeutic interventions [6], [7]. Despite decades of research toward developing vaccines against *Schistosoma japonicum* (*S.japonicum*), however, a protective vaccine against this pathogen is still not available.

A potential issue limiting the immune system’s response to vaccination is the presence of regulatory T cells (Tregs) which suppress T cell activation [8], [9]. Tregs play a central role in immune homeostasis and in preventing autoimmune disease. Natural Tregs which express Foxp3 and antigen-specific Tregs which secrete IL-10 and/or TGF-β, termed Tr1 or Th3 cells, play a protective role in immunity to infection by controlling infection-induced immunopathology [10]. However, induction of Tregs to suppress the host’s protective immune responses is also a potent immune subversion strategy utilized by many pathogens, including *S. japonicum*, to prolong their survival [11], [12]. Both thymus-derived natural Tregs and pathogen-induced peripheral Tregs could contribute to the immune suppression observed during infection [13]. Depletion of these natural and induced Tregs, consequently, can enhance the development of protective T cell responses during chronic infection [14], [15].

Studies have demonstrated that vaccination may also lead to the expansion of CD4^+^CD25^+^ Tregs, which ultimately blunts responses to cancer vaccines. Indeed, the depletion of this cell type results in an enhanced tumor vaccination response [16], [17]. The potent immunosuppressive effects of CD4^+^CD25^+^ Tregs may in part explain the failure of many immunotherapeutic approaches to cancer [18], [19]. For example, treatment with cyclophosphamide to reduce suppressor cells has been shown to enhance antitumor immunity during vaccination in melanoma patients. However, it is now recognized that more specific strategies are required to eliminate Tregs in order to improve the efficacy of anti-tumor immunotherapeutics [20]. A common method of depleting CD25^+^ Tregs is to inject an antibody against CD25, which is constitutively expressed on this cell type. This approach has been demonstrated to significantly improve the clearance of injected tumor cells [16], [21]. A similar strategy is required to enhance the efficacy of poorly immunogenic prophylatic infectious disease vaccines and for therapeutic vaccination in chronic infections [22], [23]. Although previous studies point to the importance of CD4^+^CD25^+^ Tregs in the host response to cancer and other diseases, the influence of these cells on the response to a schistosomiasis vaccine has yet to be examined.

A 26 KDa isoenzyme of *S.japonicum* glutathione S-transferase (Sj26GST), which catalyses detoxification of lipophilic molecules by thioconjugation, is one of the six antigens recommended by WHO for vaccine development [24]. It has been shown that the reduction of worm burdens and liver egg numbers in mice can reach 30.1% and 44.8%, respectively, after immunization with a plasmid containing Sj26GST DNA (pVAX1-Sj26GST) [25]. In order to investigate the influence of CD4^+^CD25^+^ Tregs on the response to a schistosome vaccine, this study evaluated whether the depletion of CD4^+^CD25^+^ Tregs using anti-CD25 antibody treatment leads to an enhancement of pVAX1-Sj26GST DNA vaccine potency in mice. The results demonstrated that CD25^+^ cell depletion did not enhance protection conferred by pVAX1-Sj26GST vaccination, but did cause a significant increase in splenocyte proliferation and IgG levels. Depletion of CD25^+^ cells induced splenic CD4^+^CD25^−^ T cell secretion of both IFN-γ and IL-10, which may, in part, explain the lack of enhancement of the protection conferred by vaccines.

## Results

### Anti-CD25 Monoclonal Antibody Treatment Depletes Treg Cells in C57BL/6 Mice Prior to pVAX1-Sj26GST Vaccination

**Figure 1:**
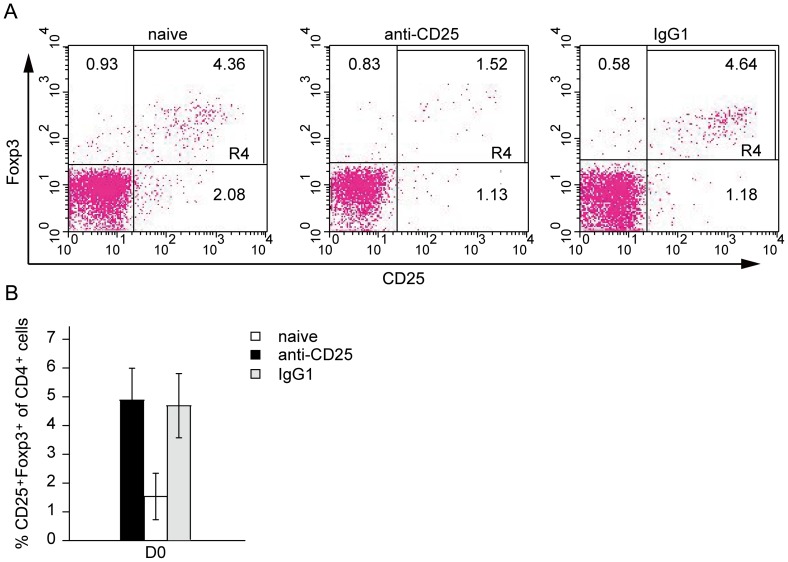
Effective depletion of CD4^+^CD25^+^ T cells in mice treated with anti-CD25 antibody. C57BL/6 mice (6 per group) were intraperitoneally administered anti-CD25 mAb or rat isotype control IgG1 in 500 µg/mouse on day -3. Peripheral blood was obtained by retro-orbital bleed, red blood cells were excluded on day 0, and samples were then subjected to flow cytometry analysis for CD3, CD4, CD25 and Foxp3. (A) Representative flow cytometry data indicating the percentages of CD4^+^CD25^+^Foxp3^+^ Tregs in the peripheral blood of mice treated with anti-CD25 mAb. Double-staining for CD25 and Foxp3 expression in cells gated for CD3^+^ and CD4^+^. Values indicate the percentage of events in the indicated quadrant. (B) Bar graph depicting the percentages of CD4^+^CD25^+^Foxp3^+^ Tregs isolated from the peripheral blood of mice treated with anti-CD25 or isotype IgG1. The data are expressed as the mean values of two experiments with three mice per group.

Given the potential role of CD4^+^CD25^+^ Treg cells in suppressing the immune response induced by vaccination, a key question is whether the depletion of this cell type affects the protective efficacy of the pVAX1-Sj26GST schistosomiasis vaccine. To deplete CD4^+^CD25^+^ Treg cells, C57BL/6 mice were administered a 500 µg/mouse dose of the anti-CD25 PC61 antibody via intraperitoneal injection. This treatment protocol has previously been shown to deplete and inhibit CD4^+^CD25^+^ Tregs [23]. As CD25 is expressed on effector T cells generated upon immunization, as well as on CD4^+^CD25^+^ Tregs, it is not possible to examine the effect of the anti-CD25 antibody on Tregs by surface phenotype. The transcription factor Foxp3 is associated with CD4^+^CD25^+^ Tregs identity and function. Therefore, co-expression of CD25 and Foxp3 was used to identify CD4^+^CD25^+^ Tregs [26]. The effectiveness of the treatment regimen was confirmed by FACS analysis of peripheral blood from either control rat IgG1 or anti-CD25 mAb treated mice (Figure 1A). Three days after treatment, compared to control mice, anti-CD25 treatment resulted in an average reduction of 66% in CD4^+^CD25^+^Foxp3^+^ Treg cell number (Figure 1B).

### Pre-emptive Depletion of CD25^+^ Cells does not Significantly Improve the Protective Efficacy of pVAX1-Sj26GST Vaccination

**Figure 2:**
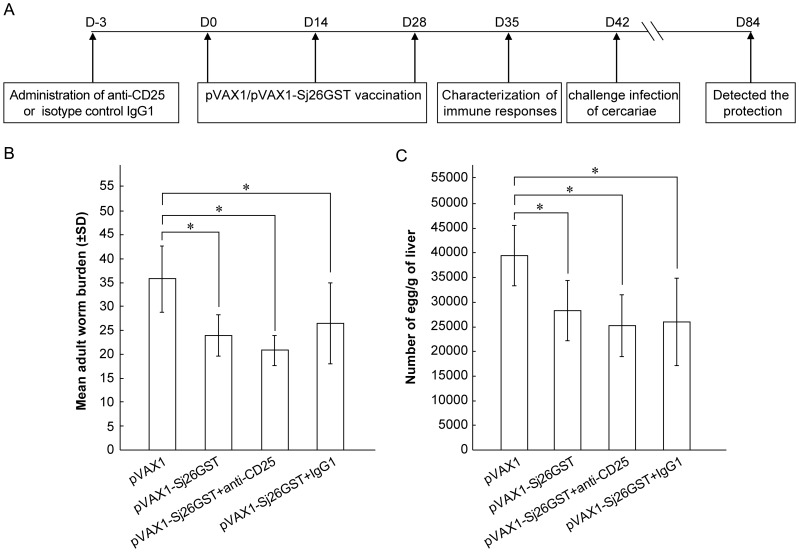
CD25^+^ cell depletion does not increase protection induced by immunization with pVAX1-Sj26GST. C57BL/6 mice (6 per group) were intraperitoneally administered 500 µg/mouse of anti-CD25 or isotype IgG1 on day -3. Three days later (D0), the mice were vaccinated with 50 µg/mouse of pVAX1 or pVAX1-Sj26GST DNA intradermally with three doses on a two weeks interval. Seven days after the last dose of vaccine, mice were sacrificed for the characterization of cellular and humoral immune response. Alternately, two weeks after the final vaccination, mice (8 per group) from each group were challenged percutaneously with 40±1 *S. japonicum* cercariae. Six weeks later the mice were sacrificed and perfused to determine worm burdens and liver egg burdens. (A) Experimental model diagram. The adult worm (B) and liver egg burdens (C) per mouse in each group were determined. The data are expressed as the mean±SD (n = 8) and are representative of two independent experiments. *P<0.05.

To assess the effect of CD4^+^CD25^+^ Treg depletion on the protective efficacy of the pVAX1-Sj26GST vaccine, C57BL/6 mice were subjected to anti-CD25 antibody treatment, or treated with rat IgG1 antibody or no antibody as controls, and immunized intramuscularly three days later (day 0) with 50 µg pVAX1 or pVAX1-Sj26GST DNA. The treatment regimen is illustrated in Figure 2A. The percentage of protection induced by vaccination was measured by the reduction in adult worm and egg burden. Among mice with no antibody pre-treatment, those inoculated with pVAX1-Sj26GST show a reduction in worms of 33.23%, and a reduction of eggs in the liver of 28.42% (*P*<0.05), compared with the pVAX1 inoculated control group (Figures 2B and 2C). Similarly, mice pre-treated with the control IgG1 antibody and inoculated with pVAX1-Sj26GST show a 26.15% reduction in worms and a 34.21% reduction of eggs in the liver (*P*<0.05) compared to control inoculation. However, pre-treatment with anti-CD25 antibody followed by vaccination with pVAX1-Sj26GST results in a slightly higher reduction in worm burden (41.82%) and liver egg reduction (36.24%) compared to control inoculated mice (Figures 2B and 2C). This indicates that anti-CD25 antibody treatment does not significantly improve the protective efficacy of pVAX1-Sj26GST vaccination.

### Kinetics and Characterization of Treg Cell Induction during pVAX1-Sj26GST Vaccination

**Figure 3:**
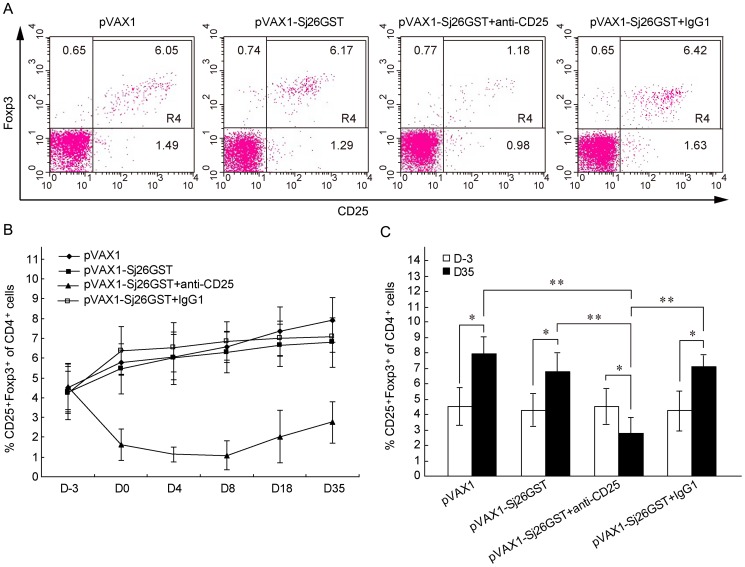
Peripheral Treg cells are induced during pVAX1-Sj26GST vaccination. (A) Seven days after anti-CD25 mAb administration, peripheral blood was obtained by retro-orbital bleed, red blood cells excluded and samples were subjected to flow cytometry analysis for CD3, CD4, CD25 and Foxp3. Representative flow cytometry data demonstrates the percentages of CD4^+^CD25^+^Foxp3^+^ Tregs in the peripheral blood of mice treated with anti-CD25 mAb at D4 in each of the treatment groups. (B) Percentage of CD4^+^CD25^+^Foxp3^+^ Tregs in peripheral blood samples on the indicated days after vaccination. Groups consisted of three mice at each time point. The results are representative of two independent experiments. (C) Bar graph depicting the percentages of CD4^+^CD25^+^Foxp3^+^ Tregs from the peripheral blood of mice treated with anti-CD25 or isotype IgG1 on D-3 and D35. Groups consisted of six mice at each time point. The results are expressed as the mean±SD of 12 mice from two independent experiments. *P<0.05; **P<0.01.

This limited change in disease protection conferred by pVAX1-Sj26GST vaccination after CD25^+^ cell depletion may be explained by Tregs that remain or recover after antibody treatment. It has been previously reported that the depletion of CD4^+^CD25^+^ Tregs with anti-CD25^+^ treatment is not completely effective [27]. Tracking the kinetics of CD4^+^CD25^+^ Treg cell numbers after immunization and CD25^+^ cell depletion demonstrates that depletion is effective 3 days after injection (day 0), reaches a maximal level of a 70–80% reduction on day 8, and remains significantly lower than that of pre-immunization on day 35. Further, after immunization, the percentage of CD4^+^CD25^+^Foxp3^+^ Tregs in CD25^+^ cell-depleted mice after vaccination with pVAX1-Sj26GST was significantly lower than that in other groups (Figure 3). However, both pVAX1 and pVAX1-Sj26GST-immunized mice had significantly increased percentages of CD4^+^CD25^+^ Tregs after vaccination, compared to before vaccination, suggesting that vaccination induced production of peripheral Treg cells. Overall, these data suggest that CD25^+^ cell recovery after depletion likely does not explain the limited disease protection elicited by the vaccine in CD25^+^ depleted mice.

### Depletion of CD25^+^ Cells Enhances Splenocyte Proliferation and the Production of IgG Antibody after Vaccination with pVAX1-Sj26GST

**Figure 4:**
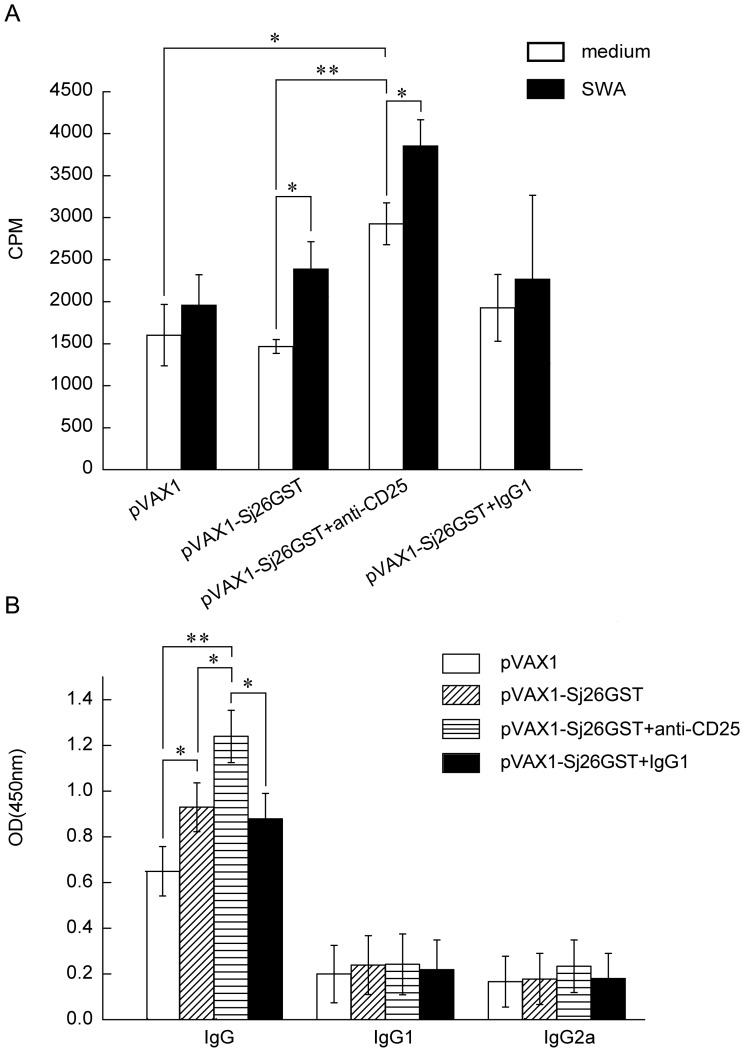
Enhancement of splenocyte proliferation and antibody production in CD25^+^ cell depleted mice. (A) Seven days after the last immunization with pVAX1 or pVAX1-Sj26GST, splenocytes were harvested and antigen-specific proliferation was measured**.** Splenocytes (2×10^5^/well) from each mouse were incubated in triplicate for three days in 200 µl in 96-well plates in the presence of SWA (15 µg/ml), or control media. 0.5 µCi [^3^H] thymidine was added to each well 16 h before the end of the incubation period. Data are expressed as the mean±SD (n = 6 per group) and are representative of three independent experiments performed in triplicate wells. *P<0.05; **P<0.01. (B) IgG, IgG1, and IgG2a responses in immunized mice. Antibody responses to SWA (15 µg/ml) were determined by ELISA. Data are expressed as the mean±SD (n = 6 per group) of 18 mice from three independent experiments performed in triplicate wells. *P<0.05; **P<0.01.

CD4^+^CD25^+^ Tregs have specifically been shown to suppress the immune response to schistosome infection [12], [28], [29]. We investigated whether CD25^+^ cell depletion *in vivo* would allow a more robust induction of immune responses after pVAX1-Sj26GST vaccination. To determine the influences on the immune response following antigen specific stimulation, splenocyte cell proliferation and antibody production were assessed. Splenocytes were isolated from CD25^+^ cell-depleted and non-depleted pVAX1-Sj26GST vaccinated mice, pooled, and stimulated with soluble worm antigen (SWA). Only Treg-depleted mice produce splenocytes that vigorously proliferate in the absence of *in vitro* stimulation with SWA (Figure 4A), suggesting that pVAX1-Sj26GST vaccination induces T cell activation *in vivo* after CD4^+^CD25^+^ Treg cell depletion. Furthermore, *in vitro* SWA stimulation causes a significant increase in splenocyte proliferation in both CD25^+^ cell-depleted mice and controls after pVAX1-Sj26GST vaccination (Figure 4A). This suggests that pVAX1-Sj26GST immunization induced antigen-specific T-cell proliferation, regardless of CD25^+^ cell depletion.

To examine whether the depletion of CD25^+^ cells influences antibody production, the levels of specific SWA antibodies in the serum of CD25^+^ cell-depleted mice after pVAX1-Sj26GST immunization were examined. Among non-CD25^+^ cell-depleted mice, pVAX1-Sj26GST vaccination causes a significant increase in antigen-specific IgG levels (*P*<0.05) compared with control inoculation (Figure 4B). However, after cell depletion, pVAX1-Sj26GST vaccination causes an even more robust increase in IgG response (*P*<0.05) than in non-depleted vaccinated mice. No IgG1 or IgG2a response was observed in immunized mice, regardless of CD25^+^ cell depletion (Figure 4B). Taken together, these results indicate that CD25^+^ cell depletion specifically influences both the proliferation of splenocytes and IgG production.

### CD25^+^ Cell Depletion Prior to Vaccination Upregulates Both Pro- and Anti-inflammatory Cytokines in pVAX1-Sj26GST-vaccinated Mice

**Figure 5:**
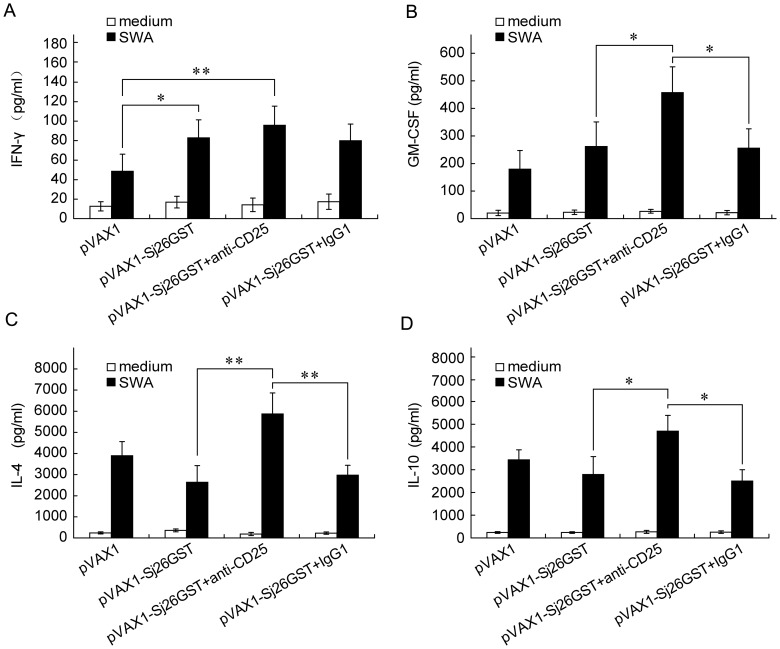
CD25^+^ cell depletion prior to DNA vaccination upregulates both pro- and anti-inflammatory cytokines in pAVX1-Sj26GST-vaccinated mice. Seven days after the last immunization with pVAX1 or pVAX1-Sj26GST, splenocytes (2×10^5^/well) from each mouse were incubated in triplicate wells for three days in 200 µl of media in 96-well plates in the presence of SWA (15 µg/ml), or control media. Supernatants were collected after 72 h of culture and tested for IFN-γ (A), GM-CSF (B), IL-4 (C), or IL-10 (D). Bars show the mean±SD (n = 6 per group) of 18 mice from three independent experiments performed in triplicate wells. *P<0.05; **P<0.01.

To further investigate the influence of CD25^+^ cell depletion on the immune response, the levels of cytokines in splenocytes isolated from CD25^+^ cell-depleted, pVAX1-Sj26GST-vaccinated mice after SWA stimulation were examined. pVAX1-Sj26GST vaccination significantly increases the production of IFN-γ and GM-CSF in all cases (*P*<0.05; Figures 5A and 5B), while IL-4 and IL-10 levels are not significantly changed in vaccinated control Ab-treated mice (Figures 5C and 5D). When CD25^+^ cells are depleted prior to immunization, IFN-γ levels in splenocyte supernatants are not increased to a higher level than that observed in non-CD25^+^ cell-depleted mice (*P*>0.05; Figure 5A). However, GM-CSF, IL-4, and IL-10 are significantly increased after immunization of CD25^+^ cell-depleted mice, compared to non-depleted controls (*P*<0.05; Figure 5C–D). Overall, these results demonstrate that CD25^+^ cell depletion prior to pVAX1-Sj26GST vaccination causes the upregulation of both pro- and anti-inflammatory cytokines.

### CD4^+^CD25^−^ T Cells from pVAX1-Sj26GST Vaccinated-mice after CD25^+^ Cell Depletion are a Major Source of Increased IFN-γ and IL-10

**Figure 6:**
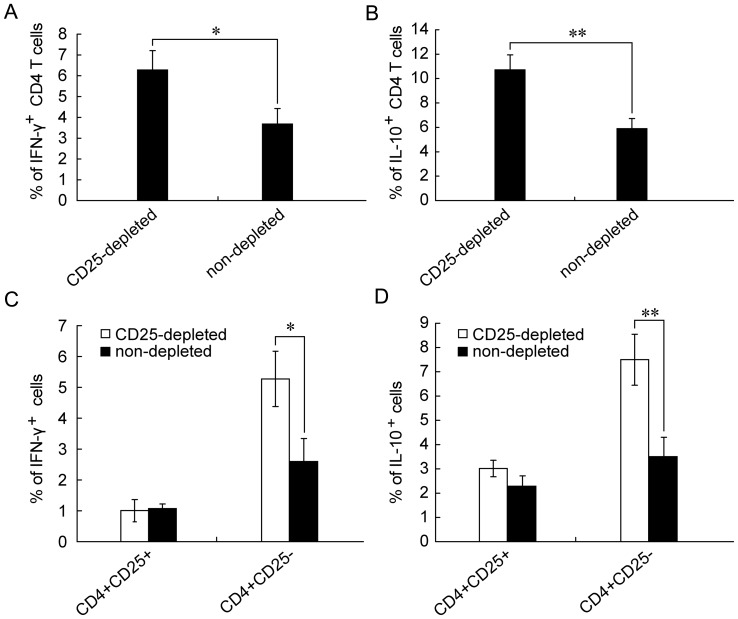
CD4^+^ and CD4^+^CD25^−^ T cells are the major source of IFN-γ and IL-10 in CD25^+^ depleted mice. Seven days after the last immunization with pVAX1-Sj26GST, splenocytes were harvested and intracellular IFN-γ and IL-10 expression were analyzed by flow cytometry. The cells were stimulated with PMA, ionomycin, and GolgiStop for 4 h at 37°C in complete RPMI 1640 media. Cells were then incubated with anti-CD3-PerCP, anti-CD4-FITC and anti-CD25-APC mAbs, washed, fixed and permeabilized with Cytofix/Cytoperm solution. Cells were stained intracellularly with anti-IFN-γ-PE, anti-IL-10-PE, or rat IgG1 (isotype control) for FACS analysis. The percentage of IFN-γ(A) and IL-10 (B) in the CD4^+^ T cells were gated on the CD3 and CD4 population. The percentage of IFN-γ (C) and IL-10 (D) in the CD4^+^CD25^+^ or CD4^+^CD25**^−^** T cells were gated on the CD3, CD4 and CD25 population. Data are expressed as the mean±SD (n = 6 per group) and are representative of two independent experiments performed in triplicate wells. *P<0.05; **P<0.01.

IFN-γ produced by CD4^+^ Th1 cells is known to be a key cytokine in promoting schistosome vaccine-induced protection, while IL-10 is a key inhibitor of the process [30]. Furthermore, as the CD4^+^ T-cell mediated immune response plays a central role in the control of schistosoma after natural infection or vaccination [6], [30], we determined whether CD4^+^ T cells are responsible for the production of these two cytokines. Splenocytes were isolated from pVAX1-Sj26GST-vaccinated mice with and without CD25^+^ cell depletion, labeled for the surface markers CD3, CD4, and CD25, and also intracellular IFN-γ and IL-10, and analyzed by flow cytometry. Compared to those isolated from non-depleted mice, CD4^+^ T cells from CD25^+^ cell-depleted mice show a significant increase in secretion of both IFN-γ (6.28±0.93% versus 3.67±0.75%; p = 0.019) and IL-10 (10.71±1.22% versus 5.89±0.83%; p = 0.005) (Figures 6A–B). However, gating CD25^+^ cells from splenocyte isolates after CD25^+^ cell depletion reveals that CD4^+^CD25^−^ T cells produce significantly higher levels of both IFN-γ and IL-10 than CD4^+^CD25^+^ cells (*P*<0.05 and *P*<0.01; Figures 6C–6D). These results indicate that CD4^+^CD25^−^ T cells are one of the major sources of both IFN-γ and IL-10 after CD25^+^ cell depletion.

## Discussion

CD4^+^CD25^+^ Tregs affect the potency of vaccines with respect to both vaccine-induced self-antigen and foreign-antigen immune responses in multiple systems [31]. A number of groups have found that depletion of CD25^+^ cell populations using an anti-IL-2 receptor alpha chain antibody (anti-CD25 antibody) [32] potentiates vaccine-induced immunity to both tumors [16], [21] and pathogens [23], [33]. However, the impact of CD4^+^CD25^+^ Tregs on vaccines against schistosomiasis, a disease that poses a significant public health concern in many tropical countries, was unknown, and is the subject of this investigation.

In the current study, to investigate the impact of CD4^+^CD25^+^ Treg cells on vaccines against schistosomiasis, we have chosen a plasmid encoding Sj26GST as a DNA vaccine. Sj26GST is recognized as a promising vaccine candidate against *S. japonicum* [6]. However, like other vaccine candidates, the current schistosoma vaccine induced limited protection, highlighting the possible negative influence of Treg cells (e.g. CD4^+^CD25^+^ Tregs ) in response to the vaccine. Indeed, the present study demonstrates that pVAX1-Sj26GST immunization induces a significant increase of CD4^+^CD25^+^Foxp3^+^ Tregs, which may be involved in the limited protection the vaccine confers. This finding is consistent with a recent publication showing that Sj26GST vaccine can enhance the expression of CD4^+^CD25^+^ Tregs in the infected animals, resulting in poor disease protection [34]. Whether other candidate antigens could induce CD4^+^CD25^+^ Tregs after immunization requires further analysis. Although not many schistosoma vaccines that elicit Treg cell development upon vaccination have been characterized [34], our group and others have demonstrated that several schistosoma antigens can induce CD4^+^CD25^+^ Tregs [12], [35]. Therefore, it is not surprising that many schistosoma antigens do not confer protection as recombinant proteins [6]. Whether these antigens could induce CD4^+^CD25^+^ Tregs upon immunization also requires further analysis.

Regarding protective immunity, CD25^+^ cell depletion prior to vaccination with the *S. japonicum* pVAX1-Sj26GST DNA vaccine results in a significant increase in vaccine-induced splenocyte proliferation and IgG levels. However, CD25^+^ cell depletion does not significantly enhance the disease protection conferred by the vaccine. These observations imply that there might be other factors that affect the vaccine efficacy after CD25^+^ cell depletion. Furthermore, these results appear inconsistent with previous studies, as a number of groups have shown that removal of CD4^+^CD25^+^ Tregs with anti-CD25 treatment enhances both the immune response and therapeutic potency of vaccines [16], [17], [23], [36], [37]. However, consistent with the current work, Tuve and colleagues report that CD4^+^CD25^+^ Tregs depletion is inefficient in controlling tumor growth in a mouse model of cervical cancer [38]. Even though CD25^+^ cell depleted mice challenged with rotavirus had improved antigen-specific CD4^+^ and CD8^+^ T cell responses, the clinical outcome was not improved [33]. Whether these differences are due to different host system, different disease models, or different vaccine formulations remains to be investigated in future studies.

Quantification of cytokines in splenocyte culture supernatants indicates that pVAX1-Sj26GST vaccination induces significant levels of IFN-γ and low levels of IL-4 and IL-10 in vaccinated control Ab-treated mice. However, immunization induces significantly higher levels of IL-4, IL-10, as well as GM-CSF in CD25^+^ cell-depleted mice. A high elicited ratio of IFN-γ/IL-10 is predictive of the success of certain vaccines [39], [40]. It is known that activation of CD4^+^ Th1 cells stimulates the production of a high level of IFN-γ, which promotes protective immune responses, and of IL-10, which plays a negative role in the development of immunity against *S,japonicum* [6]. Previous studies report that CD25^+^ cell depletion results in a significant increase in IFN-γ production in splenocyte culture supernatants, decreases the production of IL-10 in viral infection, and enhances the specific immune response induce by viral infection [33]. In contrast, this study found that CD25^+^ cell depletion causes an increase in both pro-inflammatory cytokines (e.g. IFN-γ, GM-CSF and IL-4) and an anti-inflammatory cytokine (e.g. IL-10).

Although IFN-γ and IL-10 can be produced by many cell types, including B cells, macrophages, and CD4^+^ or CD8^+^ T cells [41], [42], [43], the CD4^+^ T cell mediated immune response plays a central role in the control of schistosoma after natural infection or vaccination [6], [30]. Therefore, in this study, we assayed the production of IFN-γ and IL-10 by CD4^+^ T cells. Consistent with other studies on parasitic infection [44], [45], we found that CD4^+^ T cells produce higher levels of IFN-γ and IL-10 after CD25^+^ cell depletion following pVAX1-Sj26GST immunization. Although CD4^+^CD25^+^ Tregs have been shown to secrete IL-10 [46], [47], intracellular cytokine staining analysis in this study shows that CD4^+^CD25^−^ T cells, not CD4^+^CD25^+^ T cells, produce IL-10 and IFN-γafter CD25^+^ cell depletion. Our finding that the upregulation of IFN-γ and IL-10 in CD25^+^ cell-depleted mice may explain the impaired inhibition of IL-10 and IFN-γ production by CD4^+^CD25^−^ T cells, or other cells, after CD25^+^ cell depletion. Notably, it has been demonstrated that host-protective IL-10 is produced, through autocrine signaling, by conventional IFN-γ-producing Th1 cells during infection with *Toxoplasma gondii* [48]. Certain studies have suggested that IFN-γsecretion enhances IL-10 production, particularly in disease conditions in which the host has already been primed to antigen, such as in chronic infection or cancer [49]. Antigen-experienced T cells appear to require IFN-γ to further enhance IL-10 secretion for the inhibition of antigen-specific T cell responses [49]. Whether the induction and the suppressive function of IL-10-producing CD4^+^CD25^−^ T cells in pVAX1-Sj26GST vaccination after CD25^+^ cell depletion are dependent on IL-10 *in vivo* requires further investigation. Our findings that CD25^+^ cells depletion elicits the upregulation of both IL-10 and IFN-γ in CD4^+^CD25^−^ T cells may imply that a feedback mechanism occurs after CD25^+^ cell depletion and vaccination, which may be involved in self-regulation of inflammation by anti-inflammatory cytokines (e.g, IL-10) after vaccination. This suggests that immune homeostasis shapes the delicate balance between pro- and anti-inflammatory cytokines and regulatory and effector T-cell function, in a manner corresponding to immunological threat and minimizing damage to the host.

In conclusion, this work demonstrates that depletion of CD25^+^ cells increased immune response, but did not confer enhanced protection after immunization with *S. japonicum* pVAX1-Sj26GST. Based on our interpretation of the data, the failure of CD25^+^ Treg cell depletion to enhance vaccine-mediated protection may be due to: (i) insufficient splenocyte proliferation and IgG levels to promote immune killing of the schistosomes. A complex organism, such as *S. japonicum*, needs vaccines to stimulate the appropriate immune response that leads to protection. Studies have shown that protection elicited by vaccination is not dependent on one immune mechanism, but is multifactorial, involving both cellular and humoral elements that can be affected by the host’s genetic background and the vaccine regimen [50], [51]. Although depletion of CD25^+^ cells elicits increased IgG production and T cell response, it may not induce a sufficiently wide spectrum of immune responses. (ii) complex negative regulatory strategies, such as IL-10 production by CD4^+^CD25^−^ T cells. This observation is consistent with results of a study showing a role for IL-10 in the suppression of host immunity upon vaccination; the blockade of IL-10 allowed an ineffective therapeutic DNA vaccine to stimulate even stronger immunity and enhance clearance of persistent viral replication [52]. (iii) Similar to cancer and infection, systemic immunization is able to induce antigen-specific T cells in the peripheral system, but cannot overcome the immunosuppressive microenvironment within local immune response sites. Studies have reported that numbers of CD4^+^CD25^+^ Tregs in infective or intratumoral sites were significantly increased compared to the number of peripheral blood mononuclear cells (PBMC) [53], [54]. Indeed, natural and inducible CD4^+^Foxp3^+^ Tregs are recruited in the liver after schistosoma infection, providing an essential regulatory arm that stabilizes the immune response and limits immunopathology [29]. Apart from Tregs, others cells and molecules, such as regulatory B cells, inhibitory soluble factors (e.g., TGF-β), and inhibitory cell surface receptors (e.g., FasL, PD-L1 and B7-H1) are likely to be involved in the suppression of vaccine-mediated protection, but their roles are not yet clear.

In addition, the selection of a suitable adjuvant and delivery system to aid in the stimulation of the appropriate immune response is a critical step in the path to the development of successful antischistosome vaccines. Recently, a study showed that Lipopolysaccharide (LPS) as an adjuvant can support the development of diverse CD4^+^ T cell subsets, depending on the tissue microenvironment. For example, mice sensitized, intranasally, with a low dose of LPS display heightened Th2 responses against an allergen, whereas intravenous immunization generates Treg cells that limit CD8^+^ T cell response and intraperitoneal injection leads to Th17 and Th1 expansion in small intestinal lamina propria [55]. A similar study involving *Leishmania donovani (L. donovani)* vaccination suggested that mice immunized intraperitoneally (i.p.) and intravenously (i.v.) with *L. donovani* promastigote membrane antigens (LAg), either free or encapsulated in liposomes, were protected against challenge infection with *L. donovani*, whereas mice immunized through the subcutaneous (s.c.) or intramuscular routes were not protected. The induction of high prechallenge TGF-β limits the efficacy of s.c. vaccination, rendering it nonprotective [56]. It is not yet clear whether a traditional delivery system or an adjuvant used with schistosoma vaccines could induce CD4^+^CD25^+^ Tregs upon immunization. Elucidation of the protective mechanisms of schistosoma vaccines depends on increased depth of understanding of basic immunological knowledge. We are now working to clarify the reasons for the failure of the vaccine, with or without CD25^+^ cell depletion, to elicit protective responses. We are investigating the possibilities discussed above, toward developing strategies for schistosomiasis vaccine formulation and delivery.

Although we did not demonstrate improved protective efficacy using the CD25^+^ cell depletion strategy, we did gain insight regarding effective design of *S. japonicum* vaccines. CD25^+^ cell depletion combined with the inhibition of IL-10 may represent a promising new approach for effective schistosomiasis vaccine design. Furthermore, to develop a successful schistosomiasis vaccine, we should consider immune regulation from a broader perspective, with an appreciation of interactive networks, within and beyond the immune system, that play roles in the response to vaccination.

## Materials and Methods

### Ethics Statement

Animal experiments were performed in strict accordance with the Regulations for the Administration of Affairs Concerning Experimental Animals (1988.11.1), and all efforts were made to minimize suffering. All animal procedures were approved by the Institutional Animal Care and Use Committee (IACUC) of Nanjing Medical University for the use of laboratory animals (Permit Number: NJMU 09-0128).

### Animal Studies

Six-week-old C57BL/6 female mice were provided by the Center of Experimental Animals (Nanjing University, Nanjing, China) and bred in university facilities. All animal experiments were performed in accordance with the Chinese laws for animal protection and in adherence to experimental guidelines and procedures approved by the Institutional Animal Care and Use Committee (IACUC), the ethical review committee of Nanjing Medical University, for the use of laboratory animals. *Oncomelania hupensis* harboring *S. japonicum* cercariae (Chinese mainland snail strain) were purchased from the Jiangsu Institute of Parasitic Diseases (Wuxi, China).

### DNA Vaccine Preparation

Constructs encoding the Sj26GST were prepared and confirmed as described previously [25], using the 3 kb recombinant expression plasmid pVAX1 (a gift from Professor Jiaojiao Lin, Shanghai Veterinary Research Institute, Chinese Academy of Agricultural Sciences, Shanghai, China) containing the cytomegalovirus (CMV) promoter and bovine growth homone (BGH) polyadenylation signal. Constructs were confirmed by sequencing. Expression of Sj26GST was verified by transfecting the pVAX1-SjGST plasmid into 293 cells. The empty vector pVAX1 was used as control. Plasmids were replicated in DH5α *Escherichia coli* and purified with Qiagen Endo-Free plasmid kit (Qiagen, Valencia, CA) according to the manufacturer’s protocol. The Limulus Amebocyte Lysate QCL-1000® kit (Cambrex, Charles City, IA, USA) was used to confirm that the Endotoxin concentration were below 0,1 EU (Endotoxin Units) per dose.

### Depletion of CD4^+^CD25^+^ T Cells

For the *in vivo* depletion of CD4^+^CD25^+^ T cells, mice were intraperitoneally injected with 500 µg of the anti-CD25 monoclonal antibody clone PC61 (BD Bioscience, Pharmingen, San Diego, Calif.) or a rat IgG1 isotype control (Sigma-Aldrich). Depletion efficiency was verified by staining with anti-CD25 antibody clone 7D4 (BD Bioscience, Pharmingen, San Diego, Calif.) followed by measurement with flow cytometry (see Results).

### Immunization and Challenge Infection

For characterization of immune responses, three independent experiments were carried out. In each experiment, C57BL/6 mice (6 mice per group) were intramuscularly injected in the quadriceps muscle with 50 µg of pVAX1 or pVAX1-Sj26GST. The immunization was repeated three times at 14-day intervals. One week after the final vaccination, mice were sacrificed for the characterization of their cellular and humoral immune response.

For the vaccination challenge trial, two independent experiments were carried out. In each experiment, C57BL/6 mice were divided into four groups of 8 mice per group. Three days after anti-CD25 or control antibody injection, each mouse was intramuscularly injected with 50 µg of pVAX1 or pVAX1-Sj26GST. Immunization was repeated three times at 14-day intervals. Two weeks after the final vaccination, all mice from each group were challenged percutaneously with 40±1 *S. japonicum* cercariae. After six weeks, mice were sacrificed and perfused to determine adult worm burdens and the liver egg burdens. Reductions in worms/liver egg burdens are expressed as a percentage of the burden recorded in the control groups.

### Antibody Detection in the Sera of Immunized Mice

For antibody detection, serum samples were collected seven days after the last immunization. Standard ELISAs were performed using soluble worm antigen (SWA) as the antigen source, which was prepared as previously described [25], [57], [58]. Antibody detection in the sera of immunized mice was performed as previously described [59]. In brief, ELISA plates (Titertek Immuno Assay-Plate, ICN Biomedicals Inc., Costa Mesa, CA, USA) were coated with SWA (15 µg/ml) in 50 mM carbonate buffer (pH 9.6), and stored overnight at 4°C. Each plate was washed three times with PBS (pH 7.6) containing 0.05% Tween-20 (PBST), and blocked with 0.3% (w/v) bovine serum albumin (BSA) in PBS for 1 h at 37°C. The plates were further washed three times with PBST, and then incubated with sera diluted with 0.3% BSA (1∶100) for the detection of IgG, IgG1, and IgG2a antibodies at 37°C for 1 h. The plates were washed four times with PBST, followed by incubation with HRP-conjugated rat anti-mouse IgG, IgG1, and IgG2a (1∶1000) for 1 h at 37°C. The plates were washed five times with PBST and were then developed with tetramethylbenzidine (TMB) substrate (BD Biosciences Pharmigen) for 30 min. Optical density (OD) was read at 450 nm using a BioRad (Hercules, CA, USA) ELISA reader.

### Splenocyte Proliferation Responses and Cytokines Determination

[^3^H] thymidine (^3^H-TdR) incorporation was used to measure splenocyte proliferation. Seven days after the last immunization, six mice from each group were sacrificed and splenocytes were harvested. In 96-well plates, 2×10^5^ cells per well were incubated for 72 h in 200 µl of complete media in the presence of SWA (15 µg/ml). After 56 h in culture, [^3^H] thymidine (0.5 µCi) (Amersham, Burkinghamshire, UK) was added to each well. At the end of the incubation period, the cells were harvested on filters and the incorporated [^3^H] thymidine counted.

To evaluate cytokine production, single-cell suspensions of splenocytes were cultured in the presence of 15 µg/ml SWA or control medium at 2×10^5^ cells/well in round bottom 96 well plates. After 3 days, culture supernatants were collected and assayed for IFN-γ, GM-CSF, IL-4, and IL-10 using FlowCytomix Mouse Cytokine Kit (Bender MedSystems, Vienna, Austria) according to the manufacturer’s instructions.

### Flow Cytometry

For analysis of CD4^+^CD25^+^Foxp3^+^ T cells, the Mouse Regulatory T Cell Staining Kit (eBioscience, San Diego, CA) was used. Whole blood from immunized mice was obtained retro-orbitally. RBC lysis was done on whole blood as needed with 1×ammonium chloride lysing solution (BD PharMingen) [60]. Cells were surface-stained with PerCP anti-CD3 mAbs (eBioscience, San Diego, CA), FITC anti-CD4 mAbs, and APC anti-CD25 mAbs, followed by fixation and permeabilization with Cytofix/Cytoperm. Intracellular staining with phycoerythrin (PE) mouse anti-Foxp3 or PE IgG2a rat immunoglobulin control antibody was performed according to the manufacturer’s protocol.

For the detection of intracellular cytokines, pooled spleen and LN cells from immunized mice were stimulated in the presence of PMA (25 ng/ml), ionomycin (1 µg/ml), and GolgiStop™ (0.66 µl/ml) at 2×10^6^/ml (2 ml/well) in 24-well plates for 6 h at 37°C in 5% CO_2_. The cells were then incubated with anti-CD3-PerCP, anti-CD4-FITC and anti-CD25-APC mAbs, washed, fixed and permeabilized with Cytofix/Cytoperm solution (BD PharMingen). Cells were then intracellularly stained with 0.2 mg/ml anti-IL-10 mAb, 0.2 mg/ml anti-IFN-γmAb or PE-conjugated rat IgG1 (isotype control) for 1 h at room temperature. Finally, cells were washed in PBS containing 1% FCS, and FACS analysis was performed with the FACS Calibur (Becton Dickinson, San Jose, CA).

### Statistical Analysis

Statistical analyses were performed using SPSS version 10.1 (Statistical Package for Social Sciences, Chicago, IL statistical software). Statistical significance was determined by Student’s *t*-test with *P*<0.05 considered statistically significant.
